# Enhancement of urban heat load through social inequalities on an example of a fictional city King’s Landing

**DOI:** 10.1007/s00484-016-1230-z

**Published:** 2016-08-18

**Authors:** M Žuvela-Aloise

**Affiliations:** Zentralanstalt für Meteorologie und Geodynamik (ZAMG), Vienna, Austria

**Keywords:** Urban heat island, Urban climate, Environmental justice, Social inequalities, King’s Landing, Urban green spaces, MUKLIMO_3 model

## Abstract

The numerical model MUKLIMO_3 is used to simulate the urban climate of an imaginary city as an illustrative example to demonstrate that the residential areas with deprived socio-economic conditions can exhibit an enhanced heat load at night, and thus more disadvantageous environmental conditions, compared with the areas of higher socio-economic status. The urban climate modelling simulations differentiate between orographic, natural landscape, building and social effects, where social differences are introduced by selection of location, building type and amount of vegetation. The model results show that the increase of heat load can be found in the areas inhabited by the poor population as a combined effect of natural and anthropogenic factors. The unfavourable location in the city and the building type, consisting of high density, low housing with high fraction of pavement and small amount of vegetation contribute to the formation of excessive heat load. This abstract example shows that the enhancement of urban heat load can be linked to the concept of a socially stratified city and is independent of the historical development of any specific city.

**Electronic supplementary material:**

The online version of this article (doi:10.1007/s00484-016-1230-z) contains supplementary material, which is available to authorized users.

## Introduction

High temperatures, especially long exposure to excessive heat during heat waves, are associated with severe impacts on human health and increase in mortality rates (Souch and Grimmond 2004; Tan et al. 2007; Baccini et al. 2008; Son et al. 2012). The phenomenon of the urban heat island (UHI) generates further excess heat within urban areas (Landsberg 1981; Oke 1982), which makes city residents more vulnerable to climate impacts as the global temperature increases (Gabriel and Endlicher 2011).

The energy balance in a densely built urban environment is modified by a number of factors: lack of vegetation, high absorption of solar radiation on paved surfaces, heat storage by built-up structures, trapping of long-wave radiation in urban canyons, reduced circulation of air and the additional release of anthropogenic heat (Oke et al. 1991). Large cities have a heterogeneous morphology with an uneven distribution of built-up and green areas. Different land use properties, orography, geographical position and regional climate play a role in forming the local climate. As a result, complex spatial patterns of urban heat load exist, which leave some areas of the city more exposed to excessive heat than others. The relationship between the vegetation and lower surface temperatures is well established (Gill et al. 2007; Bowler et al. 2010; Dousset et al. 2011), and the green infrastructure plays a major role in mitigating the UHI effect and improving the thermal comfort (Santamouris 2014; Zhang et al. 2014; Georgi and Dimitriou 2010; Norton et al. 2015).

In addition to counteracting the negative effects of the urban climate, urban green spaces provide a number of social and health benefits which are relevant for a higher quality of life for city residents (Chiesura 2004; Solecki et al. 2005; van den Berg et al. 2010; Astell-Burt et al. 2013; Qin et al. 2013; Kabisch and Haase 2014). Epidemiological studies show that access to green spaces has a positive influence on the longevity of urban citizens and self-reported health (Takano et al. 2002; Maas et al. 2006; Thompson et al. 2012; Besenyi et al. 2014). However, accessibility of green spaces is often highly stratified based on socio-economic factors such as income, race, ethnicity, age, gender or health conditions (Solecki and Welch 1995; Martin et al. 2004; Barbosa et al. 2007; Wendel et al. 2012; Vaughan et al. 2013; Reyes et al. 2014; Shareck et al. 2014). There is ample evidence that shows that the amount of vegetation, and in particular tree cover, is often substantively lower in areas with higher levels of socio-economic deprivation (Pauleit et al. 2005; Astell-Burt et al. 2014; Sander and Zhao 2015), which increases the risk of negative climate impacts for residents with lower income. The link between an individual’s socio-economic position and their health has been observed, and the uneven accessibility to urban green spaces has become recognised as an issue of environmental justice (Ernstson 2013; Wolch et al. 2014).

Studies have suggested several reasons why green spaces are differentially distributed within the urban landscape and why they are frequently less available to the poorer groups. These include the history of land use development, differences in the philosophy of park design, evolving ideas about leisure and recreation, and past social and political situations (Wolch et al. 2014). Green spaces are often viewed as luxury, and their inadequate valorisation leads to an inequitable distribution and access (Wendel et al. 2012; Wolch et al. 2014). Rapid urbanisation in many cases leads to an increase in built-up at the expense of green areas. City growth combined with urban planning policies to provide energy-efficient housing by densification of the residential areas can accelerate the loss of urban green spaces (Dallimer et al. 2011).

While most of the studies relate the unequal distribution of green spaces and resulting characteristics of urban climate to the development of a particular city, the question arises whether there is a general connection between the socio-economic conditions and negative environmental impacts. Moreover, there is a serious lack of knowledge in understanding possible relationships between the socio-economic system and the environmental issues, which is extremely important for development of urban planning strategies dealing with the climate change, such as mitigation or adaptation measures. This study is intended to demonstrate the link between the socio-economic inequality and differences in local climate on an abstract example in order to show that the relationship between the low socio-economic status and disadvantageous environmental conditions in urban area lies beyond the development of an individual city. An example of a virtual city, that is realistic enough to capture physical characteristics of an urban environment, but also simple enough to include only main features in development of a socially unequal city, is used. This study postulates that merely the concept of a socio-economic stratification can lead to an enhancement of heat load in socially deprived areas.

An illustrative example of an imaginary city called the King’s Landing is chosen to represent the socially unequal city. The King’s Landing is the capital of a fictional medieval land described in the series of fantasy novels *A Song of Ice and Fire* (Martin 1996–2011 ). The detailed portrayal of the city in Martin’s books and a map depicting main features of the natural landscape and urban structures (King’s Landing Map 2011) allow a precise characterisation of the urban environment. The fantasy novels are filmed in the TV series Game of Thrones with the historical city of Dubrovnik, Croatia, chosen as the filming location. Consequently, Dubrovnik is used in this study as a reference for the building structure and climate conditions. The King’s Landing is depicted as a stone-walled city located on rocky terrain near a river, surrounded by fields and forest, in a warm and dry climate. The aristocracy and religious leaders live in the fortresses in elevated areas with access to parks, while the poor population is situated in the densely built area located at the foot of the hills.

Urban climate model simulations with the spatial resolution capable of resolving the temperature gradients on a scale of building blocks are performed. The orography and land use characteristics are adjusted to fit the description of the imaginary city. The thermal stress and human comfort in summer period is analysed through calculation of perceived temperature and its diurnal variations for selected weather situations. The climate indices related to air temperature extremes, such as mean annual number of hot days and tropical nights, are used to describe the thermal conditions in urban environment on a climatological scale taking into account variety of weather conditions. The urban heat load is therefore used as a general term to describe thermal conditions in urban environment. The meteorological data of Dubrovnik are used as a base for the calculation of climate indices. The setup of the model is described in the “Methodology” section. “Results” section presents the results of the simulations. Sensitivity tests examine the influence of the orography, the natural landscape, the walls and the building distribution (uniform versus socially differentiated) on the urban climate. A discussion is provided in the “Discussion” section, and the conclusions are given in the “Conclusions” section.

## Methodology

### Urban climate model MUKLIMO_3 and evaluation of the climate indices

The urban climate model simulations are performed with the numerical model MUKLIMO_3 (in German: Mikroskaliges Urbanes KLImaMOdell in 3-Dimensionen). MUKLIMO_3 is a non-hydrostatic microscale model based on Reynolds-averaged Navier–Stokes (RANS) equations designed to simulate atmospheric flow fields in the presence of buildings (Sievers and Zdunkowski 1985; Sievers 1990; Sievers 1995). The thermo-dynamical version of the model includes prognostic equations for air temperature and humidity; the parameterisation of unresolved buildings using the porous media approach (Gross 1989); short-wave and long-wave radiation; balanced heat and moisture budgets in the soil (Sievers et al. 1983); and a vegetation model based on Siebert et al. (1992). The model considers friction effects on building surfaces and turbulence generation due to airflow separation. Turbulent fluxes of heat, moisture and momentum are calculated by a first-order closure scheme. The numerical method for the calculation of short-wave irradiances at the ground level, the walls and the roof of buildings in an environment with unresolved built-up is described in Sievers and Früh (2012). The storage of heat into the urban fabric is simulated by calculation of the molecular heat fluxes from the outer wall surfaces into the urban fabric (or vice versa) which depend on the temperature gradient across the walls of the buildings and the predefined values of the heat transfer coefficient and the heat capacity of the walls. The model takes into account the effects of cloud cover on radiation. However, it does not include cloud processes, precipitation, horizontal runoff or anthropogenic heat. The vegetation in the canopy model has three vertical layers: tree crown, tree trunk and low vegetation. Grid cells with buildings include only low vegetation.

The urban climate model MUKLIMO_3 is selected for the study because of its capability to simulate the atmospheric processes in the urban environment on a high spatial resolution scale (∼100 m), which is necessary to capture the thermal gradients within the city. The dynamical core of the MUKLIMO_3 model, based on the RANS equations, is designed for practical engineering, and the turbulence processes are parameterised with possible overestimation of the turbulent mixing. Similar simulations with more spatial detail could be performed with the microclimate models (e.g. ENVI-met, RayMan) in a resolved building environment if the modelling domain has a spatial extent large enough to capture the interaction between various land use types, and the modelling approach includes the effects of orography.

The 3D simulation is initialised and driven by a 1D version of the MUKLIMO_3 model. The 1D model calculates the daily cycle of temperature, relative humidity and wind for the reference station located outside of the urban area, taking into account the geographic position, height, soil type and land use characteristics of the reference station. At the beginning of the simulation, soil temperature and moisture are set constant throughout the soil column. The initial atmospheric profiles for air temperature, humidity and wind, as well as soil temperature and moisture are either taken from observations or tuned to fit different weather conditions in the case where idealised simulations are performed. The 1D simulation is run for 24 h after which the values for air temperature, relative humidity and wind are used to initialise the 3D model taking into account terrain height and soil type. The 1D model provides the upper boundary conditions for the entire duration of the 3D model run by providing hourly values for wind, air temperature and relative humidity at the top layer of the 3D model. The lateral sides of the 3D model have free boundary conditions with horizontal advection terms being zero at the upstream domain boundaries. Due to the high computational requirement, the 3D simulations are typically run for 24 h. The first hour of the 3D model simulation, where the applied initial vertical profiles from the 1D model are adjusted for the urban area, is excluded from the analysis. The meteorological fields given as the output of the 3D model are used for the analysis of the UHI effect and the calculation of climate indices.

**Fig. 1:**
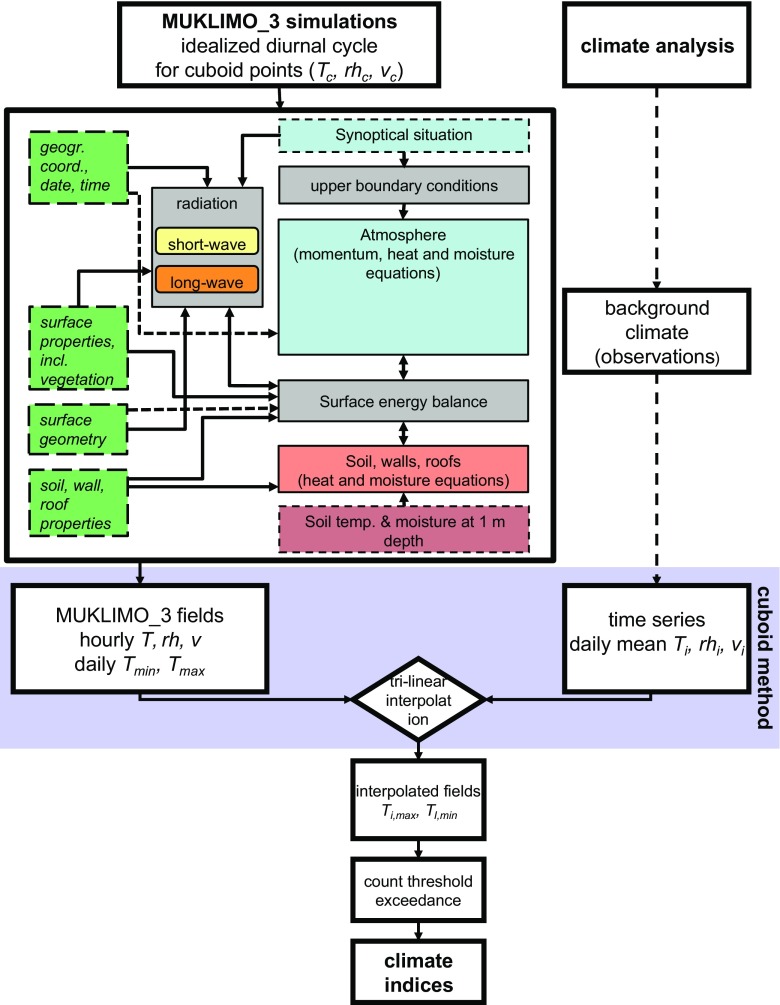
Schematic diagram of the MUKLIMO_3 model (adapted from Sievers and Jendritzky (1986); source: DWD (2015)) and the calculation of climate indices using the cuboid method (Früh et al. 2011)

In order to evaluate the mean state of urban climate, particularly the urban heat load in summer period, the climate indices, such as mean annual number of summer days (*T*_max_ ≥ 25 °C), hot days (*T*_max_ ≥ 30 °C) and tropical nights (*T*_min_ ≥ 20 °C), are used. Additionally, perceived temperature based on the Klima-Michel-Modell (Staiger et al. 2012) is calculated for the selected weather situations based on the MUKLIMO_3 model output. The climate indices are calculated with the cuboid method (Früh et al. 2011). The cuboid method enables the calculation of heat load on a longer temporal scale by using a limited number of urban climate model simulations. Instead of calculating daily simulations for the 30-year period, the 3D simulations are performed only for a selected range of weather situations where excessive heat load in the urban area can occur. Thereafter, the climate indices are derived by a tri-linear interpolation of modelled fields, which are weighted based on the real meteorological data of the mean daily air temperature (*T*), relative humidity (*rh*) and wind speed (*v*) at a reference station outside of the city. The selected weather situations can be described by a combination of the (*T*_*c*_, *rh*_*c*_, *v*_*c*_) which are named the “cuboid corners.” They have a typical range: *T*_*c*min_ = 15 °C, *T*_*c*max_ = 25 °C; *rh*_*c*min_ = 42 %, *rh*_*c*max_ = 80 %; and *v*_*c*min_ = 0.7 m/s, *v*_*c*max_ = 4 m/s. For clarification, the climatological records from Central European cities (Früh et al. 2011; Zuvela-Aloise et al. 2014) show that when the regional daily mean air temperature is below *T*_*c*min_ = 15 °C, the *T*_max_ in the city centre will hardly exceed 25 °C. Therefore, these situations can be excluded from the calculation of summer heat load, which greatly reduces the number of simulations necessary for the climate evaluation. The model work flow is illustrated in Fig. 1.

The MUKLIMO_3 model has been employed in a number of urban climate studies in German cities (Früh et al. 2011; Hoffmann et al. 2014; Buchholz and Kossmann, 2015) and evaluation of urban heat load on the climatological scale with the cuboid method was used for the cities in Central Europe (Bokwa et al. 2015). In case of Vienna, the modelling results show very good agreement with observations for the heat load during the day (Zuvela-Aloise et al. 2016), while climate indices related to minimum temperature are overestimated. The same overestimation for the nighttime conditions is not found in all modelling examples and a detailed validation of the meteorological fields in the real case applications provided by the high-resolution model remains a problem due to small number of monitoring stations, especially when climatological evaluation is concerned.

### Model setup for King’s Landing

**Fig. 2:**
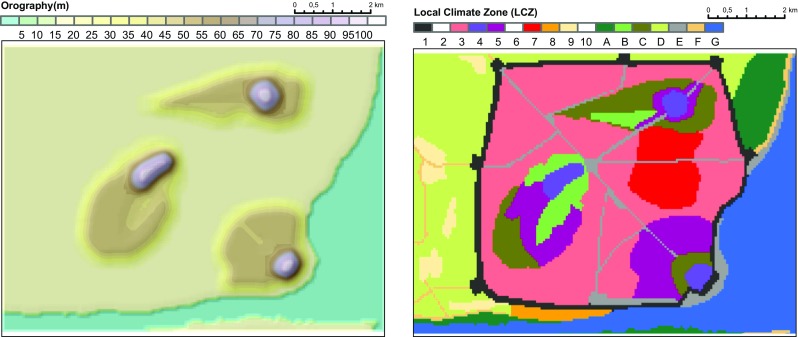
Model orography (*left*) and land use distribution (*right*). The classification of local climate zones (LCZs) is given in Table 1

The model domain covers an area of 10.1 km × 7.6 km with a domain size of 168 × 126 × 25 grid points. The horizontal grid has equidistant grid spacing of 60 m. The vertical resolution varies from 10 to 50 m with higher resolution near the ground. The model height is 510 m, while maximum elevation of the terrain is 100 m. The size of the domain is approximately ten times larger than the walled city of Dubrovnik. This adjustment is intended to match the estimated population of the imaginary city King’s Landing (ca. 500,000 inhabitants), which is approximately ten times more than the population of the city of Dubrovnik. The digitalisation scheme of the land use is similar to the method applied for the reconstruction of the urban climate of Vienna based on historical maps (Zuvela-Aloise et al. 2014). The map of the King’s Landing (King’s Landing Map 2011) is transferred into polygons, and for each polygon area, a corresponding land use class is defined (Fig. 2).

**Fig. 3:**
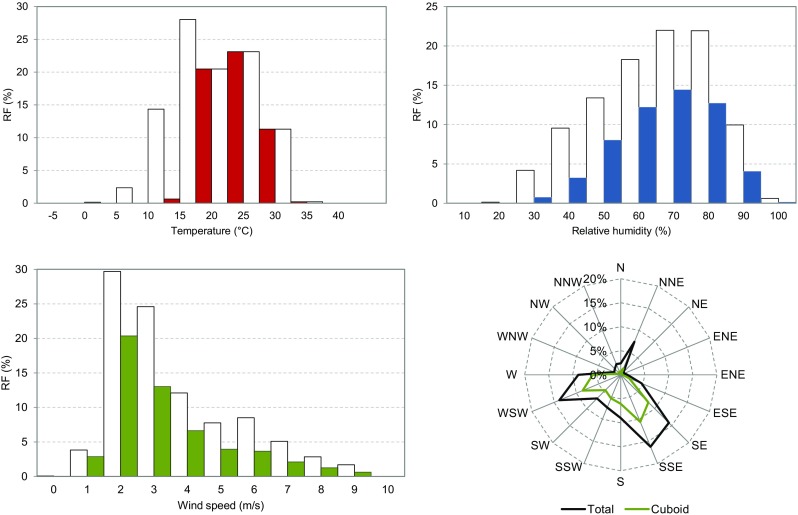
Relative frequency (RF) distribution of daily mean temperature, relative humidity, wind speed and wind direction at 1400 CET for the period 1981–2010 at the reference station Dubrovnik (lat 42° 39′ N, long 18° 05′ E, height 52 m). The *values in colour* indicate the range used in the cuboid method (*T*_*c*min_ ≥ 15 °C) (data source: DHMZ–Croatian Meteorological and Hydrological Service)

Sixteen idealised simulations with duration of 24 h for two opposite wind directions (NW and SE) and the geographical coordinates of Dubrovnik are calculated. The initial and boundary conditions for idealised simulations of the cuboid corners are given by a time-varying 1D vertical profile (up to 3360 m) of temperature and relative humidity for low and high wind speed conditions. All simulations are started on July 15 with 1 day spin-up for the 1D boundary model. The 3D simulation is started on July 16 at 0900 Central European Summer Time (CEST) and ends on July 17 at 0800 CEST. July 15 is chosen as a representative day for the calculation of summer heat load since it has an average sun height and day length for the conditions in June to August where most summer days occur. The water temperature is set to 24 °C and kept constant in all simulations. The 1D model is initialised with different stability conditions, cloudiness (from 0 to 0.875), soil moisture, soil temperature (16 and 23 °C) and indoor temperature (20 and 25 °C). The indoor temperature is constant during the simulations, and air conditioning is not included. Daily time series from a Dubrovnik monitoring station are used to calculate 30-year averages of climatic indices (Fig. 3). For each land use category, a set of model parameters is defined to describe surface properties and urban structures. The parameters include the following: fraction of built area (*γ*_*b*_), mean building height (*h*_*b*_), wall area index (*w*_*b*_), fraction of pavement of the non-built area (*v*), fraction of tree cover (*σ*_*t*_) and fraction of low vegetation of the remaining surface (*σ*_*c*_), height (*h*_*c*_) and leaf area index (*LAI*_*c*_) of the canopy layer as well as mean height (*h*_*t*_) and leaf area index (*LAI*_*t*_) of the trees with separated values for the tree trunk and the tree crown area. The land use classes and parameters are defined following the Stewart and Oke (2012) local climate zone (LCZ) classification system (Table 1). In this case, the model differentiates between 14 land use categories: 7 built-up classes, 4 vegetation types, bare surface, rocks and water. The building geometry, which is not defined by the LCZ classification system, is estimated from the building structure in Dubrovnik.

**Table 1:** Parameters for land cover properties in MUKLIMO_3 model following the Stewart and Oke (2012) local climate zone (LCZ) classification system

Local climate zone (LCZ)		*γ*_*b*_^a^ (%)	*h*_*b*_^a^ (m)	*w*_*b*_	*v* ^a^ (%)	*σ*_*t*_^a^ (%)	*σ*_*c*_^a^ (%)	*h*_*t*_ (m)	*h*_*c*_ (m)
1	Compact high-rise	0.60	25	6.67	0.40	0.0	0.01	0	0.3
2	Compact midrise	0.55	15	4.50	0.30	0.0	0.08	0	0.3
3	Compact low-rise	0.55	7	2.33	0.20	0.0	0.13	0	0.3
4	Open high-rise	0.30	25	7.00	0.35	0.0	0.25	0	0.3
5	Open midrise	0.30	15	4.50	0.35	0.0	0.21	0	0.3
6	Open low-rise	0.30	7	2.33	0.35	0.0	0.25	0	0.3
7	Lightweight low-rise	0.75	3	1.80	0.15	0.0	0.03	0	0.3
8	Large low-rise	0.40	7	1.40	0.45	0.0	0.08	0	0.3
9	Sparsely built	0.15	7	2.80	0.10	0.0	0.60	0	0.3
10	Heavy industry	0.25	10	3.00	0.30	0.0	0.14	0	0.3
A	Dense trees	0.00	0	0.00	0.00	0.8	0.18	17	0.5
B	Scattered trees	0.00	0	0.00	0.00	0.4	0.54	9	0.5
C	Bush, scrub	0.00	0	0.00	0.00	0.0	1.00	0	1.5
D	Low plants	0.00	0	0.00	0.00	0.0	1.00	0	0.5
E	Bare rock or paved	0.00	0	0.00	0.95	0.0	0.01	0	0.3
F	Bare soil or sand	0.00	0	0.00	0.00	0.0	0.01	0	0.3
G	Water	0.00	0	0.00	−1.00	0.0	0.01	0	0.3

*γ*_*b*_ fraction of built area, *h*_*b*_ mean building height, *w*_*b*_ wall area index, *v* fraction of pavement, *σ*_*t*_ fraction of tree cover, *σ*_*c*_ fraction of low vegetation, *h*_*t*_ tree height, *h*_*c*_ height of the low vegetation

^a^Relative to total grid cell area

## Results

**Fig. 4:**
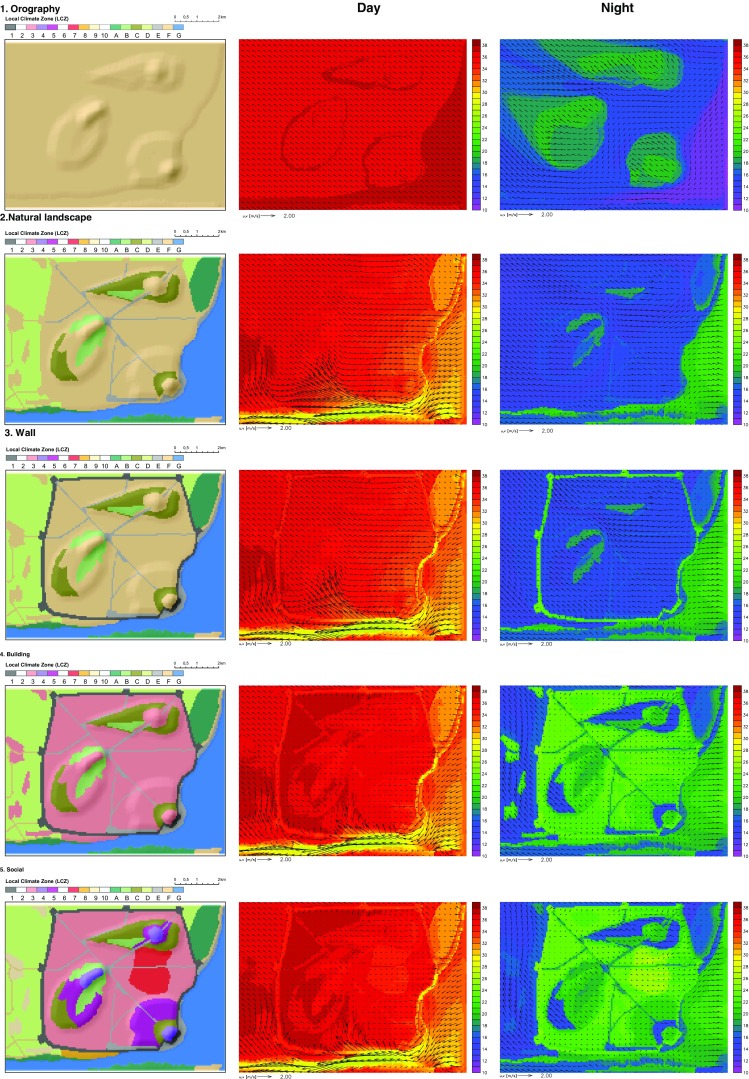
Land use distribution (*left*), perceived temperature (°C) and wind field (m/s) at the reference level (2 m) at 1600 CEST (*middle*) and 0200 CEST (*right*) in the idealised simulations with prevailing SE wind, low mean wind speeds (0.7 m/s), high mean temperature (25 °C) and low mean relative humidity (40 %)

In order to illustrate the role of different natural and anthropogenic factors in the formation of the UHI, the modelling simulations are split into five stages of gradual city development. The sensitivity scenarios include (1) orography, (2) natural landscape, (3) wall, (4) buildings and (5) social effect. Sensitivity simulations are performed by changing the land use parameters in idealised simulations for the cuboid corners. The model results for perceived temperature and wind field are shown in Fig. 4, and evaluation of thermal stress on a typical hot day, representing warm, dry, and weak wind conditions (T_cmax_ = 25 °C, rh_cmin_ = 40 %, v_cmin_ = 0.7 m/s) is given in Table A1, including statistical analysis for each LCZ.

**Fig. 5:**
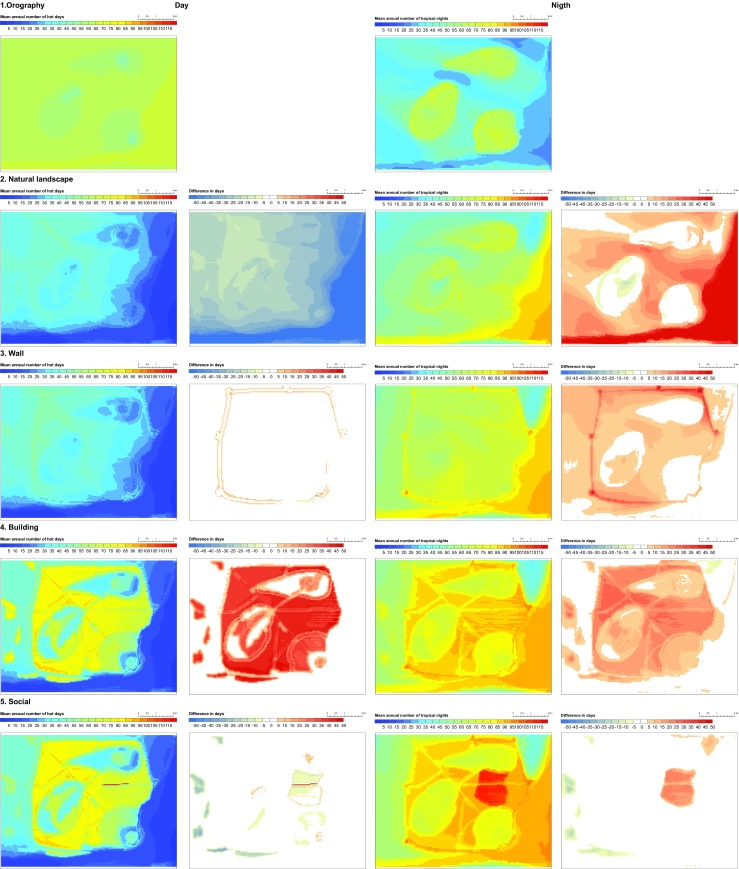
Diurnal and nocturnal urban heat load expressed in mean annual number of hot days (*T*_max_ ≥ 30 °C) and tropical nights (*T*_min_ ≥ 20 °C) with absolute values of climate indices (*left*) and difference in spatial distribution between the subsequent scenarios (*right*). Climate indices are calculated by combining the idealised MUKLIMO_3 urban climate model simulations with the meteorological data from Dubrovnik weather station for the period 1981–2010

The climate indices related to extreme air temperatures (mean annual number of hot days and tropical nights) are calculated to account different weather situations and derive robust results for the mean state of the urban climate. Spatial distribution of climate indices and the changes between the scenarios are illustrated in Fig. 5. Model results including mean values of climate indices and standard deviation for the given LCZ, as well as difference between the scenarios and the statistical significance of change are given in Table A2. General overview of the modelling results is given in Table 2.

**Table 2:** Main effects of changes in land use and building structure on the heat load

Scenario		Day	Night
1	Orography	• High heat load • Spatially uniform distribution in flat terrain • Slightly lower in elevated areas	• Low heat load • Temperature inversion in vertical • Higher in elevated areas
2	Natural landscape	• Decrease due to vegetation and water surfaces • Formation of land-sea gradient • Higher in agricultural areas • Lower near water surfaces	• Increase in low terrain • Reversal of land-sea gradient • Lower in agricultural areas • Higher near water surfaces
3	Wall	• Increase in wall area • Shadowing effect	• Strong increase in wall area • Increase in areas below the height of the wall due to reduction of ventilation
4	Building	• Strong increase in built-up areas	• Strong increase in built-up areas • Minor increase in surrounding areas due to advection of heat
5	Social	• Decrease in built-up areas with more vegetation • Minor decrease in built-up area with high density due to shadowing effect	• Minor decrease in built-up areas with low density and more vegetation • Strong increase in built-up areas with high building density and less vegetation

### Orographic effect

In the simulation where only features of terrain are considered, the land surface is elevated 20 m above the sea level and the relief is flat except for the three hills with the maximum height of 100 m. The surface is characterised as bare soil (LCZ F) with 1 % of low vegetation (0.3-m height). No water surfaces are present.

**Fig. 6:**
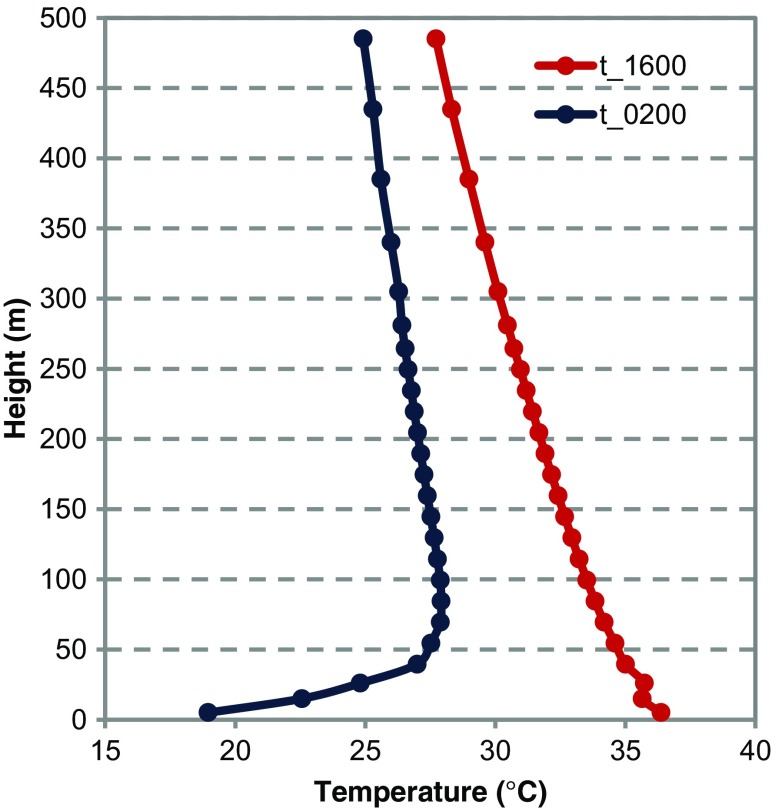
Vertical profile of air temperature for daytime (t_1600) and nighttime conditions (t_0200) in idealised simulation with prevailing SE wind, low mean wind speeds (0.7 m/s), high mean temperature (25 °C) and low mean relative humidity (40 %) in the orography scenario

The results show a uniform distribution of high temperatures during the day in the low terrain and lower temperatures on the hill sides. During the night, strong cooling of the free surface results in temperature inversion, which is visible in the vertical profile of temperature (Fig. 6). Due to the temperature inversion, elevated areas are warmer.

### Natural landscape effect

In this scenario, a natural surface cover is introduced to the orography. The land types include forest (LCZ A), scattered trees or urban park (LCZ B) and scrubs (LCZ C) located on the hillsides, agricultural fields (LCZ D) surrounding the city, bare rocks (LCZ E) and the sea (LCZ G). The remaining area is defined as free surface (LCZ F). The roads are included in the simulation since they are categorised as bare rocks.

**Fig. 7:**
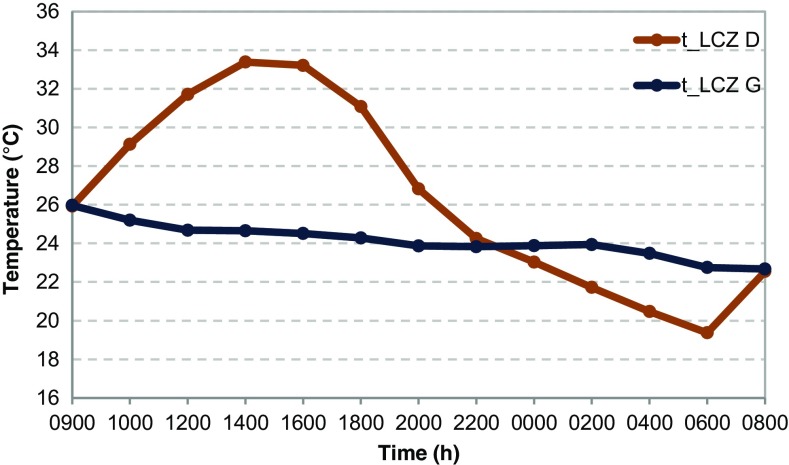
Diurnal cycle of air temperature at the reference level (2 m) for agricultural surfaces (LCZ D) and water areas (LCZ G) in idealised simulation with prevailing SE wind, low mean wind speeds (0.7 m/s), high mean temperature (25 °C) and low mean relative humidity (40 %) in the natural landscape scenario

The simulated temperatures are in general lower than in the simulation where only orography is considered. Due to different properties of surface cover, horizontal temperature gradients are formed. During the day, agricultural areas warm faster than the water areas (Fig. 7) and the circulation from the sea towards inland is enhanced, bypassing the hills as obstacles. During the night, the water areas cool slower than the land, and thus, the temperature gradient is eventually reversed with areas near the sea having higher temperatures.

### Wall effect

In the third scenario, the walls surrounding the city are introduced. The walls are defined as compact high-rise (LCZ 1) with 60 % of buildings (maximum value defined for this land use type in the LCZ classification system) and 25-m height (maximum height of Dubrovnik city walls). The MUKLIMO_3 model parameterises buildings as a porous media with lower porosity for higher building density. Therefore, the flow is reduced, but not completely blocked in presence of buildings.

The effect of the wall on the temperature distribution is most pronounced in the vicinity of the walls. During the day, a minor shadowing effect is visible, producing higher temperatures on the inner side of the western and northern walls. During the night, the temperatures along the walls are the highest and the overall area of the city within the walls is warmer, especially in the low terrain due to the reduced ventilation. However, since the walls are only 25 m high, compared to 100-m height of the hills, they do not significantly affect the elevated areas.

### Buildings effect

Free surface areas within the walls and near the roads are replaced by buildings having identical building geometry. The typical building type in Dubrovnik (Table 1) most resembles the compact low-rise (LCZ 3) with three-story buildings. The LCZ 3 building type is characterised by a high fraction of built-up area (55 %) and pavement (20 %) and a small fraction of low vegetation (13 %). There are no trees simulated on the built-up surfaces.

The modelling results show that the presence of buildings reduces ventilation and increases heat load in all areas of the city. During the day, the most pronounced heat excess is found in the low terrain in the lee-side of the hills and surrounded by walls, especially in the areas furthest from the sea. This resembles the pattern of the natural landscape on which the buildings have been superimposed. During the night, the heat load in the flat terrain is mostly uniform. Elevated and green areas have relatively lower heat loads both during the day and night. Minor, but statistically significant, change in heat load can be found in the areas of the city that remained unmodified. In this case, the heat load is increased through advection of the air masses between the neighbouring surfaces.

### Social effect

In the final scenario, the building structure has been modified to fit the social conditions described in the books. Three new building types are introduced within the city: open high-rise (LCZ 4) to characterise high towers and religious buildings, open mid-rise (LCZ 5) for the surrounding large buildings and lightweight low-rise (LCZ 7) as a densely built-up area of the slum with single-story buildings. The individual buildings for LCZ 7 are lower (3 m) than typical LCZ 3 buildings (7 m) and have a smaller surface area (50 m^2^, compared to 150 m^2^). The fraction of buildings is higher (75 %), the pavement area is lower (15 %) and the fraction of low vegetation is smaller (3 %). High-class buildings (LCZ 4 and 5) are higher than LCZ 3 buildings having heights of 25 and 15 m, respectively. The surface area of individual buildings is four to five times larger than LCZ 3 buildings, the fraction of buildings is lower (30 %) and the area has 20–25 % fraction of low vegetation.

Changing the building structure induces minor changes on the heat load during daytime conditions. The model simulations show a local increase of heat load in elevated areas for high-rise buildings (LCZ 4), but a minor decrease in the low terrain (LCZ 7) and on the elevated areas (LCZ 5). During the night, the heat load in elevated areas is locally reduced compared to the simulation with the equal buildings. However, the heat load is considerably higher in the slum area in the low terrain. Since no trees are considered in any built-up areas, the results are mostly dependent on the building geometry and relative proportions of built-up, low-vegetated and paved area.

## Discussion

The modelling simulations of the gradual development of an imaginary city are intended to illustrate the formation of spatial gradients in urban heat load due to the natural factors and anthropogenic modifications. The effects of the main physical processes in urban environment, such as absorption of solar radiation, reduction of ventilation, shadowing effect, heat storage, influence of green and water surfaces, are demonstrated in a simplified form through the different modelling scenarios. Each scenario helps to understand processes behind individual land use change and shows tendencies in heat load induced by the land use modification. The summary presented in Table 2 can be used as a simple guidance for urban planners.

In the case of the imaginary city, it is not possible to validate the modelling results directly. However, the model values for climate indices are compared with the mean annual number of hot days and tropical nights in the city of Dubrovnik. Only one climate monitoring station in the area of Dubrovnik is available, and it is located in the suburban environment in the coastal region. The mean annual number of hot days for the time period 1981–2010 amounts to 29.3, which is comparable to the modelling results for the LCZ C of 34.8 (Table A2). The observed mean annual number of tropical nights is 78.3, which is similar to the modelled value of 72.3. The use of land use classification to describe urban structures increases model uncertainties related to the choice of land use parameters, such as characteristic building density and height, fraction of pavement, pervious surfaces and vegetation. The LCZ classification system was introduced to limit and standardise the selection of the land use parameters. However, the range of parameters defined by Stewart and Oke (2012) allows for variations within one LCZ, which can lead to minor changes in quantitative evaluation of the heat load. When calculating atmospheric processes in the urban environment, the MUKLIMO_3 model takes into account not only the physical characteristics of the urban structure but also elevation and advection of the air masses between the neighbouring surfaces. The heat load for an individual LCZ depends on the location in the city, relief and the surrounding land use types. Therefore, the quantitative results for the heat load for an individual LCZ cannot be directly compared with the observations from an arbitrary city. The abstract example should be used to demonstrate general tendencies in urban heat load modification, and the quantitative values are valid within the range of model uncertainties and data used in the simulations.

An imaginary city serves as an illustrative example that does not deal with any actual city, but the concept of the socially unequal city. The modelling results point out that the excessive heat load can be formed in the socially unprivileged areas and that this might not be merely a coincidence, but a result of the general expectations how a socially stratified city looks like. For example, the climate modelling results might look different if the author of the book suggested that the area of the poor had broad green alleys, lot of parks and water surfaces, or was located on a more favourable position in the city. However, this would not resemble an image of a slum. Instead, the fantasy books focus on the social relationships and described physical characteristics are an attempt to make the city look plausible. Environmental processes and related climatic consequences are not explicitly considered in the books nor are intentional; they result from the expected social conditions.

## Conclusions

The study shows the potential development of the UHI during the summer period in the imaginary city of King’s Landing using an urban climate model MUKLIMO_3 and meteorological data for the city of Dubrovnik, Croatia. The model results show that the excessive heat load can be found in the areas inhabited by the poor population as a combined effect of natural and anthropogenic factors. Dense, low-rise, built-up areas in low terrain have higher temperatures in comparison to elevated areas with higher buildings, lower building density and more vegetation. The spatio-temporal variability of the heat load pattern is formed by both natural landscape and human modifications. During the day, the temperatures are highest inland, which can be associated with the natural characteristics of the land-sea distribution. During the night, the temperature gradient is reversed, with higher temperatures towards the sea. However, within the city walls, the building characteristics dominate with the area of the socially disadvantageous residents exhibiting the highest heat load due to unfavourable location, high building density and low amount of vegetation.

The modelling simulations show that there are two factors which contribute to the formation of the heat load in socially unprivileged areas: (1) unfavourable location, in this case location under the hill in the low terrain surrounded by the high city walls, which prevent good ventilation, and (2) the building type, consisting of high density, low housing with high fraction of pavement and less vegetation. The UHI effect is less pronounced in the elevated areas which have naturally better conditions and, additionally, less building density and more vegetation. In an abstract sense, the concept of a socially stratified city suggests that the areas that are naturally advantageous are likely to be inhabited by the higher classes and maintain good quality due to careful planning. In contrast, the less favourable areas are expected to be inhabited by lower social classes and the quality can further be deteriorated by inappropriate planning and lack of mechanisms that could compensate for the naturally disadvantageous conditions. The study shows a basic mechanism that relates lower social standards with negative environmental impacts. In this sense, the King Landing’s effect is defined as the enhancement of heat load in an urban environment by socio-economic stratification.

This study is intended to demonstrate the relationship between the low socio-economic status and disadvantageous environmental conditions on an abstract example in order to help urban planners to identify the problems and develop appropriate urban planning strategies. In the case of cities where the green spaces are predominantly available to the high-income population and value of green infrastructure is regulated through the real-estate prices, the general incentives for urban greening, as a strategies to adapt to climate change, might not necessarily lead to the desired outcome. Instead, the result can even be a gentrification and a displacement of the residents for which the green spaces were intended (Wolch et al. 2014). Therefore, urban planning strategies should consider the socio-economic structure, in order to appropriately address the environmental problems. Using the mapping of urban climate and physically based models, it is possible to identify the critical zones in which the intervention are necessary and to which extent they need to be applied to improve the environmental conditions prior to undertaking large infrastructural investments. Together with empirical research of benefits of such measures (Bowler et al. 2010) and taking into account socio-demographic status, user preferences, cultural perceptions and barriers (Kabisch and Haase 2014; Wendel et al. 2012), this information could help to develop appropriate solutions and equalise the social and environmental disparities.

In reality, the socio-economic system and the urban climate are more complex than shown in this study. The expected deprivation of urban green spaces in low-income neighbourhoods is not always found (Abercrombie et al. 2008), and even an overall increase in urban green spaces is reported in many cities (Kabisch and Haase 2013). Additionally, the relationship between green space and change in health is not always clear (Wolfe et al. 2014) and a better understanding is needed (Lachowycz and Jones 2013). The background climate conditions and the terrain morphology can modify the heat load characteristics for different LCZs. Therefore, the simple and somewhat exaggerated example presented in this study should not be used for urban planning purposes without considering the particularities of the individual city.

Nevertheless, the study points out an important mechanism that might be present in many cities but is masked by the complex dynamics of the city as a system. Understanding relationships between different elements of the system and raising awareness are primary steps in addressing environmental and socio-economic problems related to climate change. Using popular media or social games are an effective way in addressing the public at large and can help people to communicate the trade-offs between mitigation and adaptation measures in an urban environment (Juhola et al. 2013). For this reason, the popular, but instructive, example can be used for educative purposes in order to increase the interest in problems of climate change in urban environments.

## Electronic supplementary material

Table A1 (DOCX 27 kb)

Table A2 (DOCX 30 kb)
