# Supplementary material for: Enhancement of urban heat load through social inequalities on an example of a fictional city King’s Landing
Source: Int J Biometeorol. 2016 Aug 18;61(3):527–39. doi: 10.1007/s00484-016-1230-z (PMC5334419; doi:10.1007/s00484-016-1230-z)
Supplement: Supplementary file 1 — (DOCX 27 kb) [file 484_2016_1230_MOESM1_ESM.docx]

**Table A1** Perceived temperature (°C) at 1600 CEST (pt_1600) and 0200 CEST (pt_0200) in different model scenarios. The mean value and the standard deviation are given in the first row for each local climate zone (LCZ). In the second row are shown the minimum and maximum value within the LCZ. Note that the area (A) of the LCZ can differ between the scenarios. Values marked in green color indicate statistically significant change compared to the previous scenario. Statistical significance was determined by Kolmogorov–Smirnov to a 99% confidence interval.

| **Scenario** | | | **Orography** |  |  | **Natural landscape** | |  | **Wall** |  |  | **Buildings** |  |  | **Social** |  |
| --- | --- | --- | --- | --- | --- | --- | --- | --- | --- | --- | --- | --- | --- | --- | --- | --- |
|  | **LCZ** | A (ha) | pt_1600 | pt_0200 | A (ha) | pt_1600 | pt_0200 | A (ha) | pt_1600 | pt_0200 | A (ha) | pt_1600 | pt_0200 | A (ha) | pt_1600 | pt_0200 |
| **1** | Compact high-rise |  |  |  |  |  |  | 370 | 33.4 ± 1.9 | 21.9 ± 1.1 | 370 | 33.4 ± 1.8 | 22.7 ± 1.2 | 370 | 33.4 ± 1.8 | 22.7 ± 1.2 |
|  |  |  |  |  |  |  |  |  | 27.4 - 35.8 | 17.0 - 24.8 |  | 27.2 - 35.5 | 18.4 - 25.5 |  | 27.1 - 35.6 | 18.3 - 25.5 |
| **3** | Compact low-rise |  |  |  |  |  |  |  |  |  | 3239 | 36.6 ± 0.8 | 22.3 ± 1.0 | 2008 | 36.8 ± 0.7 | 22.6 ± 0.8 |
|  |  |  |  |  |  |  |  |  |  |  |  | 29.1 - 38.2 | 18.9 - 24.2 |  | 32.6 - 38.1 | 19.1 - 24.9 |
| **4** | Open high-rise |  |  |  |  |  |  |  |  |  |  |  |  | 130 | 36.1 ± 0.8 | 20.5 ± 0.6 |
|  |  |  |  |  |  |  |  |  |  |  |  |  |  |  | 33.8 - 37.4 | 19.4 - 22.2 |
| **5** | Open midrise |  |  |  |  |  |  |  |  |  |  |  |  | 549 | 36.5 ± 0.7 | 20.6 ± 0.5 |
|  |  |  |  |  |  |  |  |  |  |  |  |  |  |  | 32.8 - 37.7 | 18.7 - 21.9 |
| **7** | Lightweight low-rise |  |  |  |  |  |  |  |  |  |  |  |  | 315 | 34.6 ± 0.3 | 25.1 ± 0.9 |
|  |  |  |  |  |  |  |  |  |  |  |  |  |  |  | 33.5 - 35.3 | 21.8 - 26.3 |
| **8** | Large low-rise |  |  |  |  |  |  |  |  |  |  |  |  | 69 | 35.0 ± 1.5 | 20.3 ± 0.5 |
|  |  |  |  |  |  |  |  |  |  |  |  |  |  |  | 29.8 - 37.5 | 19.6 - 21.7 |
| **9** | Sparsely built |  |  |  |  |  |  |  |  |  |  |  |  | 167 | 37.0 ± 0.5 | 16.6 ± 0.7 |
|  |  |  |  |  |  |  |  |  |  |  |  |  |  |  | 35.2 - 37.9 | 15.6 - 19.0 |
| **A** | Dense trees |  |  |  | 440 | 32.0 ± 1.1 | 18.1 ± 1.7 | 440 | 32.0 ± 1.1 | 18.2 ± 1.7 | 440 | 32.0 ± 1.0 | 18.2 ± 1.7 | 440 | 32.1 ± 1.0 | 18.2 ± 1.7 |
|  |  |  |  |  |  | 29.0 - 37.2 | 15.3 - 21.4 |  | 28.9 - 36.9 | 14.4 - 21.5 |  | 29.0 - 36.5 | 15.2 - 21.7 |  | 29.0 - 36.8 | 15.0 - 21.6 |
| **B** | Scattered trees |  |  |  | 206 | 35.8 ± 0.3 | 18.1 ± 0.9 | 206 | 35.8 ± 0.3 | 18.4 ± 0.6 | 206 | 35.8 ± 0.3 | 19.6 ± 0.3 | 206 | 35.8 ± 0.3 | 19.5 ± 0.3 |
|  |  |  |  |  |  | 34.8 - 36.5 | 16.1 - 19.1 |  | 34.8 - 36.5 | 16.7 - 19.1 |  | 34.9 - 36.7 | 18.8 - 20.5 |  | 34.9 - 36.7 | 18.6 - 20.4 |
| **C** | Bush, scrub |  |  |  | 413 | 35.7 ± 0.8 | 15.2 ± 0.3 | 413 | 35.8 ± 0.8 | 15.3 ± 0.2 | 413 | 36.0 ± 0.8 | 15.7 ± 0.3 | 413 | 36.0 ± 0.8 | 15.7 ± 0.3 |
|  |  |  |  |  |  | 33.0 - 37.1 | 14.3 - 15.8 |  | 33.1 - 37.2 | 14.7 - 16.1 |  | 33.6 - 37.7 | 15.0 - 16.4 |  | 33.6 - 37.7 | 14.9 - 16.9 |
| **D** | Low plants |  |  |  | 1192 | 36.6 ± 0.6 | 14.3 ± 0.4 | 1192 | 36.6 ± 0.6 | 15.0 ± 0.6 | 1192 | 36.6 ± 0.6 | 15.5 ± 0.6 | 1192 | 36.6 ± 0.6 | 15.3 ± 0.7 |
|  |  |  |  |  |  | 34.3 - 37.6 | 13.5 - 15.2 |  | 34.3 - 38.2 | 13.3 - 16.7 |  | 34.6 - 38.3 | 13.5 - 17.1 |  | 34.6 - 37.9 | 13.4 - 17.0 |
| **E** | Bare rock or paved |  |  |  | 303 | 33.6 ± 1.7 | 16.4 ± 0.6 | 303 | 33.8 ± 1.5 | 16.6 ± 0.4 | 303 | 34.3 ± 1.9 | 17.6 ± 0.9 | 303 | 34.4 ± 1.9 | 17.7 ± 0.9 |
|  |  |  |  |  |  | 30.5 - 36.2 | 14.6 - 19.4 |  | 30.3 - 36.8 | 15.7 - 19.3 |  | 30.3 - 37.4 | 16.3 - 19.8 |  | 30.3 - 37.4 | 16.2 - 20.2 |
| **F** | Bare soil or sand | 7620 | 36.9 ± 0.2 | 16.1 ± 2.3 | 3736 | 35.2 ± 1.3 | 15.1 ± 0.5 | 3366 | 35.3 ± 1.2 | 15.4 ± 0.3 | 127 | 35.1 ± 1.5 | 15.6 ± 0.4 | 127 | 35.1 ± 1.5 | 15.4 ± 0.5 |
|  |  |  | 36.2 - 37.5 | 10.0 - 19.9 |  | 29.1 - 37.4 | 13.6 - 16.2 |  | 29.7 - 37.9 | 14.4 - 16.4 |  | 31.4 - 37.8 | 14.8 - 17.0 |  | 31.3 - 37.4 | 14.6 - 17.0 |
| **G** | Water |  |  |  | 1330 | 30.7 ± 1.6 | 19.8 ± 1.8 | 1330 | 30.9 ± 1.8 | 19.6 ± 1.7 | 1330 | 31.0 ± 1.9 | 19.7 ± 1.8 | 1330 | 31.0 ± 1.9 | 19.7 ± 1.8 |
|  |  |  |  |  |  | 26.5 - 34.8 | 15.9 - 22.0 |  | 27.1 - 34.6 | 15.4 - 21.9 |  | 27.1 - 34.9 | 15.5 - 22.3 |  | 27.1 - 35.0 | 15.5 - 22.4 |
