# Supplementary material for: Enhancement of urban heat load through social inequalities on an example of a fictional city King’s Landing
Source: Int J Biometeorol. 2016 Aug 18;61(3):527–39. doi: 10.1007/s00484-016-1230-z (PMC5334419; doi:10.1007/s00484-016-1230-z)
Supplement: Supplementary file 2 — (DOCX 30 kb) [file 484_2016_1230_MOESM2_ESM.docx]

**Table A2** Mean annual number of hot days (HD) and tropical nights (TR) in different model scenarios. The mean value and the standard deviation for each climate zone (LCZ) are given in the first row. Mean difference between the scenarios calculated over the area (A) of the LCZ and the standard deviation is given in the second row. Values marked in green color indicate statistically significant change compared to the previous scenario. Statistical significance was determined by Kolmogorov–Smirnov to a 99% confidence interval.

| **Scenario** | | | **Orography** |  |  | **Natural landscape** | |  | **Wall** |  |  | **Buildings** |  |  | **Social** |  |
| --- | --- | --- | --- | --- | --- | --- | --- | --- | --- | --- | --- | --- | --- | --- | --- | --- |
|  | **LCZ** | A (ha) | HD | TR | A (ha) | HD | TR | A (ha) | HD | TR | A (ha) | HD | TR | A (ha) | HD | TR |
| **1** | Compact high-rise |  |  |  |  |  |  | 370 | 33.5 ± 9.9 | 76.3 ± 7.2 | 370 | 37.8 ± 11.7 | 83 ± 8.7 | 370 | 37.4 ± 11.6 | 83.5 ± 8.7 |
|  |  |  |  |  |  |  |  |  | 0.4 ± 2.8 | 22.4 ± 9.0 |  | 4.3 ± 3.7 | 6.3 ± 4.2 |  | -0.4 ± 1.0 | 1.0 ± 2.2 |
| **3** | Compact low-rise |  |  |  |  |  |  |  |  |  | 3239 | 71.0 ± 12.0 | 83 ± 6.4 | 2008 | 75.3 ± 9.7 | 87.9 ± 4.4 |
|  |  |  |  |  |  |  |  |  |  |  |  | 34.2 ± 8.9 | 17.7 ± 6.0 |  | -0.1 ± 1.0 | 2.0 ± 1.0 |
| **4** | Open high-rise |  |  |  |  |  |  |  |  |  |  |  |  | 130 | 50.5 ± 7.2 | 73.9 ± 2.5 |
|  |  |  |  |  |  |  |  |  |  |  |  |  |  |  | 3.6 ± 2.8 | 2.1 ± 0.5 |
| **5** | Open midrise |  |  |  |  |  |  |  |  |  |  |  |  | 549 | 57.5 ± 8.5 | 76.8 ± 2.8 |
|  |  |  |  |  |  |  |  |  |  |  |  |  |  |  | -3.3 ± 2.0 | -1.3 ± 1.0 |
| **7** | Lightweight low-rise |  |  |  |  |  |  |  |  |  |  |  |  | 315 | 67.7 ± 6.0 | 107.5 ± 4.8 |
|  |  |  |  |  |  |  |  |  |  |  |  |  |  |  | -6.0 ± 4.0 | 19.0 ± 2.9 |
| **8** | Large low-rise |  |  |  |  |  |  |  |  |  |  |  |  | 69 | 47.8 ± 15.0 | 79.0 ± 4.7 |
|  |  |  |  |  |  |  |  |  |  |  |  |  |  |  | 14.0 ± 7.6 | -5.6 ± 3.5 |
| **9** | Sparsely built |  |  |  |  |  |  |  |  |  |  |  |  | 167 | 49.6 ± 7.4 | 58.3 ± 2.4 |
|  |  |  |  |  |  |  |  |  |  |  |  |  |  |  | -20.1 ± 6.6 | -11.1 ± 4.9 |
| **A** | Dense trees |  |  |  | 440 | 15.8 ± 1.9 | 47.9 ± 11.9 | 440 | 16.0 ± 2.1 | 52.5 ± 11.9 | 440 | 16.1 ± 2.3 | 54 ± 13.5 | 440 | 16.0 ± 2.2 | 55.8 ± 12.5 |
|  |  |  |  |  |  | -46.8 ± 2.5 | 12.4 ± 10.2 |  | 0.2 ± 0.5 | 4.6 ± 3.1 |  | 0.1 ± 0.7 | 1.2 ± 2.7 |  | -0.1 ± 0.3 | 2.1 ± 2.2 |
| **B** | Scattered trees |  |  |  | 206 | 33.4 ± 2.1 | 58.0 ± 5.6 | 206 | 33.9 ± 2.2 | 62.0 ± 3.0 | 206 | 39.5 ± 4.6 | 71 ± 2.2 | 206 | 39.4 ± 4.6 | 71.9 ± 2.0 |
|  |  |  |  |  |  | -24.4 ± 2.1 | 4.7 ± 5.7 |  | 0.5 ± 0.2 | 4.0 ± 3.1 |  | 5.6 ± 3.2 | 8.6 ± 2.9 |  | -0.1 ± 0.6 | 1.2 ± 0.9 |
| **C** | Bush, scrub |  |  |  | 413 | 29.7 ± 4.8 | 61.8 ± 3.5 | 413 | 30.3 ± 5.0 | 65.0 ± 2.4 | 413 | 34.9 ± 7.1 | 71 ± 3.0 | 413 | 34.8 ± 6.9 | 72.3 ± 3.4 |
|  |  |  |  |  |  | -27.9 ± 4.3 | -0.6 ± 3.8 |  | 0.7 ± 0.5 | 3.2 ± 2.1 |  | 4.6 ± 3.6 | 6.1 ± 1.7 |  | -0.1 ± 0.5 | 1.2 ± 1.2 |
| **D** | Low plants |  |  |  | 1192 | 36.9 ± 5.2 | 46.4 ± 4.8 | 1192 | 38.3 ± 5.6 | 56.0 ± 3.8 | 1192 | 40.3 ± 6.5 | 60 ± 5.8 | 1192 | 39.5 ± 6.3 | 58.8 ± 5.6 |
|  |  |  |  |  |  | -25.4 ± 5.2 | 10.4 ± 5.9 |  | 1.4 ± 1.9 | 9.6 ± 4.1 |  | 2.0 ± 1.9 | 3.6 ± 3.1 |  | -0.8 ± 1.3 | -0.8 ± 3.1 |
| **E** | Bare rock or paved |  |  |  | 303 | 31.1 ± 10.4 | 63.8 ± 9.3 | 303 | 32.6 ± 10.7 | 71.4 ± 6.5 | 303 | 48.1 ± 24.7 | 82 ± 3.6 | 303 | 48.8 ± 26.2 | 83.9 ± 4.5 |
|  |  |  |  |  |  | -30.8 ± 10.6 | 18.2 ± 11.0 |  | 1.4 ± 2.9 | 7.6 ± 4.7 |  | 15.5 ± 16.1 | 10.4 ± 5.6 |  | 0.7 ± 5.5 | 2.2 ± 2.2 |
| **F** | Bare soil or sand | 7620 | 62.1 ± 3.2 | 41.8 ± 12.6 | 3736 | 35.2 ± 7.3 | 56.9 ± 7.2 | 3366 | 36.5 ± 7.3 | 65.7 ± 4.9 | 127 | 29.1 ± 13.9 | 69 ± 10.8 | 127 | 27.8 ± 11.5 | 68.3 ± 12.8 |
|  |  |  |  |  |  | -25.9 ± 6.9 | 11.4 ± 9.3 |  | 1.1 ± 1.7 | 8.5 ± 5.2 |  | 1.9 ± 5.1 | 4.1 ± 3.6 |  | -1.2 ± 3.6 | -1.0 ± 3.9 |
| **G** | Water |  |  |  | 1330 | 9.2 ± 2.6 | 84.1 ± 5.7 | 1330 | 9.3 ± 2.4 | 86.6 ± 3.7 | 1330 | 9.6 ± 2.5 | 90 ± 3.9 | 1330 | 9.6 ± 2.5 | 91.3 ± 4.4 |
|  |  |  |  |  |  | -57.6 ± 2.4 | 55.8 ± 7.7 |  | 0.2 ± 0.5 | 2.5 ± 2.8 |  | 0.2 ± 0.3 | 3.0 ± 1.6 |  | 0.0 ± 0.2 | 1.7 ± 0.8 |
